# Somatic Symptoms: Association Among Affective State, Subjective Body Perception, and Spiritual Belief in Japan and Indonesia

**DOI:** 10.3389/fpsyg.2022.851888

**Published:** 2022-04-11

**Authors:** Venie Viktoria Rondang Maulina, Masao Yogo, Hideki Ohira

**Affiliations:** ^1^Department of Cognitive and Psychological Sciences, Nagoya University, Nagoya, Japan; ^2^Department of Psychology, Atma Jaya Catholic University of Indonesia, Jakarta, Indonesia; ^3^Department of Psychology, Doshisha University, Kyoto, Japan

**Keywords:** somatic symptoms, health concerns, trait anxiety, positive affect, negative affect, somatosensory amplification, spirituality

## Abstract

This study aimed to examine differences in the following somatic symptoms: affective state (i.e., health concerns, anxiety, and positive and negative affect), somatosensory amplification, spirituality in Japan and Indonesia, and associations among all variables from each culture. Previous studies and a potential bio-psycho-spiritual model has identified the association of each variable in the development of somatic symptoms. Moreover, they demonstrated that individuals who describe themselves as more religious and spiritual report better physical and mental health. A total of 469 and 437 university students from Japan and Indonesia, respectively, completed the questionnaires for assessing somatic symptoms, health concerns, trait anxiety, positive and negative affect, somatosensory amplification, and spiritual belief. This study found significant differences in health concerns, positive and negative affect, state anxiety, and spiritual belief. Moreover, the difference in somatosensory amplification was negligible. There is a shared association in both cultures among somatic symptoms, affective state, subjective body perception, and spirituality. Health concerns and trait anxiety moderated somatosensory amplification in the development of somatic symptoms. However, the role of spirituality belief in somatic symptoms was observed in the Japanese and Indonesian cultures in relation to positive affect.

## Introduction

Somatic symptoms are a frequent indication of emotional distress with or without a physiological basis. People frequently complain of headaches, chest pain, elevated heart rate, and other symptoms visit a general practitioner or medical facility. Moreover, somatic symptoms are key features of somatization disorder or somatic symptom disorder, where depression disorder can potentially manifest into somatic complaints (American Psychiatric Association, 2000, 2013). Such complaints differ from psychosomatic symptoms, such as duodenal ulcers or hypertension, which can be detected physiologically (Kawanishi, 1992).

Somatic symptoms tend to be influenced by biological, psychological, and social factors as well as spirituality. The biopsychosocial disease model provides a framework for understanding the complex interrelationship between spirituality and somatic symptoms (Engel, 2012). Moreover, the gate control/neuro-matrix theory of pain (Melzack and Wall, 1965; Melzack, 1999) describes the influence of biological, psychological, and social factors on the pain experience of an individual through pathways descending from the brain. The theory acknowledges bidirectionality in the relationship of pain with cognition, emotion, and behavior (Rush et al., 2020). The biopsychosocial model and gate control/neuro-matrix theory identify psychosocial variables as potential mediators and moderators of the pain experience. As such, previous studies identify several psychosocial mediators, such as mood, anxiety, social support, self-efficacy, and coping strategies (Rush et al., 2020).

Essential affective state factors in the development of somatic symptoms are health concerns or health anxiety. It manifests as disproportionate and persistent thoughts about the seriousness of one’s somatic symptoms, persistently high levels of anxiety about health or bodily symptoms, and excessive time or energy devoted to such symptoms (American Psychiatric Association, 2013). Studies that investigated the psychological factors associated with somatic symptoms claim that such symptoms were significantly correlated with overall subjective distress like negative affect—but not positive affect (Watson et al., 1988). Specifically, individuals with high scores in negative affect complain of frequent somatic symptoms (Pennebaker, 2000). Research also establishes a link between anxiety, especially health anxiety, and somatic symptoms (Maulina, 2017). This link suggests that a negative mood state may inhibit immune function and, as a result, increase vulnerability to disease and elicit somatic complaints (Leventhal et al., 1996). The relationship between somatic symptoms and emotions is correlational and may be causal to a certain degree (Pennebaker, 2000).

Moreover, somatosensory amplification plays a significant role in the emergence of somatic symptoms, where empirical findings support its association with negative affect. Somatosensory amplification refers to the tendency to experience somatic and visceral sensations as unusually intense, noxious, and disturbing (Barsky et al., 1988). It involves with hypervigilance or heightened attentional focus on bodily sensation. Scholars report that the link between somatosensory amplification and somatic symptoms exerts medium to high overall strength (Köteles and Witthöft, 2017; Ishii, 2019).

The study on spirituality/religiosity has become considerable in this context, because its relationships among health variables exist. In the bio–psycho-spiritual model, potential pathways between spirituality and pain, which are revealed as spiritual beliefs, may correlate with psychosocial and physiological changes (Wachholtz et al., 2007). Many studies indicate that spirituality is linked to increased pain tolerance, muscle relaxation, positive mood, spiritual health, spiritual experiences, and decrease in anxiety (Rush et al., 2020). Moreover, research reveals that individuals who describe themselves as religious and spiritual report being physically and mentally healthier (Koenig and Cohen, 2002; Koenig, 2012). Such belief can also affect certain neuroendocrine and immune mechanisms, which positively impact a wide variety of health outcomes, such as susceptibility to cancer and recovery after surgery in the patients (Koenig and Cohen, 2002).

Spirituality can involve cognitive or emotional states like beliefs, motivations, a sense of gratitude or attachment to God, and other spiritual thoughts and feelings. The extant literature consistently illustrates that spiritual cognitions and emotions can be further divided into positive states (e.g., faith or trust in God, secure religious attachment, and religious gratitude) and negative states (e.g., appraisals that God is punitive or unfair). The positive cognitive and emotional aspects of spirituality and religion consistently act as a buffer against anxiety (Rosmarin and Leidl, 2020). Moreover, most studies report a positive correlation between religious or spiritual involvement and increased psychological well-being, hope, optimism, purpose, and meaning to life (Koenig, 2018).

Spirituality is a complex and multidimensional issue and can be defined as an individual and open approach for searching for meaning and purpose in life (Büssing et al., 2014). Many people continue to profess their religious affiliation. However, westerners transitioned toward a less religious identity. Notably, this trend coincides with substantial increases in the prevalence and severity of mental disorders across western countries in general. Experiences described as religious, such as feeling the presence of God or a higher power, feeling guided by a Spiritual Force, being grateful for one’s blessings, and praying in its various forms, frequently occur outside the context of religion. Such spiritual experiences are common even among individuals who do not profess religious beliefs or affiliations. Nowadays, most research on religion and mental health is published using spirituality terms instead of religiosity (Rosmarin and Koenig, 2020).

According to a national survey (Japanese Institute of Statistical Mathematics, 2008), 73% of Japanese individuals do not believe in religion. However, this result does not necessarily imply that the majority of the Japanese are non-religious. Instead, it may mean that the ancient faith of Shintoism and the newly introduced religion of Buddhism have been integrated (i.e., the syncretization of Shintoism and Buddhism or *Shin*-*Butsu Shugo* in Japanese) during the long history of religion (i.e., more than 2,000 years). Faith has eventually become deeply incorporated into the psyches of the Japanese, and no longer recognized as religions (Nakao and Ohara, 2014). Many young Japanese believe in *something religious*, such as spirits and *the other world or heaven*. Commonly known as atheists, the Japanese cherish sensibility toward religious and spiritual aspects in the broader meaning of these words. A considerable segment of the Japanese population believes in invisible powers despite the lack of belief in any religion (Nishi, 2009; Nakayama, 2019). Meanwhile, Indonesia has five major religions, namely, Islam, Protestantism, Catholicism, Hinduism, and Buddhism. The majority of surveyed Indonesian residents (96%) reported a connection between their belief in God and the preservation of positive values (Tamir et al., 2020).

Both cultures may differ in terms of spiritual beliefs but have some resemblance. The previous study presents the Japanese and Indonesian cultures as adopting the interdependent model of self-construal, which consists of connectedness and relationships with others frequently observed in non-western cultures (Singelis, 1994; Park and Kitayama, 2014). People with interdependent self-construal are likely to rely on social evaluations in developing and maintaining positive self-identities (Markus and Kitayama, 1991; Park and Kitayama, 2014). When communicating the cultural norms for the expression of emotions, Asians also tend to emphasize somatic symptoms instead of emotional states (Kleinman, 1986; Choi et al., 2016; Devany and Poerwandari, 2020). Additionally, few cross-cultural studies investigated the differences in the non-western context. The current study assesses the differences between Japan and Indonesia in terms of somatic symptoms and their relationship with anxiety, somatosensory amplification, and spirituality to fill this research gap. Research also explores the psychological and spiritual factors that correlate with somatic symptoms. Hence, the current study hypothesizes that the development of somatic symptoms is linked to anxiety, somatosensory amplification, and spirituality.

## Materials and Methods

### Participants

This study was conducted in Japan and Indonesia in a non-clinical setting with the approval of the respective ethics committees. We recruited a convenience sample of 469 and 437 university students from Japan and Indonesia, respectively. The Japanese respondents consisted of 254 men (54.2%) and 215 women (45.8%) aged 18–30 years (M = 20.18 years, standard deviation [SD] = 1.41 years). Meanwhile, the Indonesian participants comprised 174 men (39.8%) and 263 women (60.2%) aged 17–27 years (M = 20.22 years, SD = 1.39 years). The data collection was accomplished before the restriction of the COVID-19 pandemic in both countries. This study followed a between-group cross-sectional design. Before the commencement of this study, the respondents provided written informed consent forms, which ensured the confidentiality of their answers and their right to withdraw from the study at any time.

### Measurement

The participants completed several questionnaires, which lasted for approximately 20 minutes. This study used the Patient Health Questionnaire Somatic Symptom Severity Scale (PHQ-15; Kroenke et al., 2002). It consists of 15 items that assess the prevalence of the most common body symptoms. The items were rated using a three-point Likert-type scale (1 = not bothered at all, 2 = slightly bothered, and 3 = extremely bothered). Cronbach’s α values were 0.844 and 0.830 for the Japanese and Indonesian versions, respectively.

The study adopted the Somatic Symptom Disorder-B Criteria Scale (SSD-12; Toussaint et al., 2015) to measure health concerns. The scale is composed of 12 items that comprise three domains or sub-criteria, namely, cognitive, affective, and behavioral, with four items each. The items were rated using a five-point Likert-type scale (0 = never; 1 = rarely; 2 = sometimes; 3 = often; 4 = very often). Cronbach’s α values were 0.872 and 0.898 for the Japanese and Indonesian scales, respectively.

The State-Trait Anxiety Inventory was used to measure the participants’ state and trait anxiety levels (STAI; Spielberger et al., 1970; Shimizu and Imae, 1981; Ginting et al., 2015). The scale comprised 40 items, which were rated using a four-point Likert-type scale (1 = not at all; 4 = very much so). For the Japanese scale, Cronbach’s α values were 0.893 and 0.852 for state anxiety (20 items) and trait anxiety (20 items), respectively. For the Indonesian scale, these values were 0.874 and 0.804 for state anxiety and trait anxiety, respectively.

To measure positive and negative affect, the brief-form of the Positive and Negative Affect Schedule (PANAS; Watson et al., 1988) was used. The scale uses 20 words to describe different feelings and emotions (10 for positive affect and 10 for negative affect). The items were rated using a five-point Likert-type scale (1 = very slightly/not at all; 2 = a little; 3 = moderately; 4 = quite a bit; 5 = extremely) to report the extent to which they experienced each feeling and emotion in the past few weeks. For the Japanese version, Cronbach’s α values were 0.877 and 0.869 for positive and negative affect, respectively. For the Indonesian version, these values were 0.862 and 0.865 for positive and negative affect, respectively.

Sensitivity to normal somatic and visceral sensations, somatosensory amplification, or bodily symptom sensitivity was assessed using the Somatosensory Amplification Scale (SAS; Barsky et al., 1988; Nakao et al., 2001), which consists of 10 self-rated statements. Items are rated using a five-point Likert-type scale (1 = not all true; 5 = extremely true). Cronbach’s α values for this scale were 0.751 and 0.815 for the Japanese and Indonesian versions, respectively.

This study used the System of Belief Inventory (SBI) to assess the religious and spiritual beliefs of the participants (Holland et al., 1998). This scale is a shortened version of the SBI-54. It evaluates religious and spiritual beliefs as a potential mediator in coping with a life-threatening illness and the measurement of quality of life. It consists of 15 items rated using a four-point Likert-type scale (0 = none of the time; 3 = all of the time for items number 2, 7, 13, and 15, and 0 = strongly disagree; 3 = strongly agree for the remaining items). Cronbach’s α values were 0.889 and 0.917 for the Japanese and Indonesian versions, respectively.

Two additional items pertaining to spirituality examine the beliefs of the participants in spiritual/religious treatment and belief in fate/destiny. Each item was considered independently to analyze the differences between the two cultures. The items were rated using a five-point Likert-type scale (0 = not at all; 5 = strongly belief).

SPSS version 27 (IBM Corporation) was used for statistical analyses. An Independent-sample t-test was performed to compare the means between variables. To measure the relationship between all variables, HAD version 17 was used for structural equation modeling (SEM) (Shimizu, 2016). The significance level of mean comparison and correlation analyses was set at *p* < 0.05. Cohen’s d was used to estimate the effect size to indicate the standardized difference between the two means. The result of Cohen’s d between 0 to 0.3 means a small effect or negligible differences.

## Results

### Differences and Associations Among Affective State, Subjective Body Perception, and Spiritual Beliefs With Regard to Somatic Symptoms

A comparison of means between the Japanese and Indonesian participants revealed significant differences in somatic symptoms and affective states (i.e., health concerns, positive affect, negative affect, and state anxiety; *p* < 0.01; Table 1). The Indonesian respondents exhibited a higher mean than the Japanese. No significant differences were observed in trait anxiety between the two cultures. Additionally, the difference in somatosensory amplification was negligible, revealing a slight difference (Cohen’s *d* = 0.231). Meanwhile, the Japanese and Indonesian respondents displayed significant differences in spiritual beliefs. The Indonesian respondents indicated a higher mean than the Japanese participants in belief in the spiritual-religious treatment and fate/destiny (*p* < 0.001; Table 1).

**TABLE 1:** Descriptive statistics of gender, somatic symptom, affective state, subjective body perception, and spiritual belief in Japanese and Indonesian participants.

	Japan (*n* = 469)		Indonesia (*n* = 437)		*t*-statistics		Cohen′s *d*
			
	Mean	SD	Mean	SD		*P*	
Gender (% women)	45.8		60.2		χ2 = 18.66	< 0.001***	Phi 0.144
PHQ > PHQ-15	19.401	4.928	22.055	5.037	–2.654	< 0.001***	–0.533
SSD > SSD-12	20.273	7.018	27.247	8.998	–6.974	< 0.001***	–0.868
PANAS PA	27.22	8.419	35.913	6.785	–8.693	< 0.001***	–1.133
PANAS NA	25.156	8.377	37.572	6.594	–12.416	< 0.001***	–1.64
STAI Y1	44.78	10.672	46.391	9.546	–1.611	0.005**	–0.159
STAI Y2	48.923	9.474	48.918	8.125	0.006	0.886	0.001
SAS	31.631	6.715	30	7.436	1.631	< 0.001***	0.231
SBI	6.919	7.185	32.874	9.07	–25.955	< 0.001***	–3.185
**Survey**							
1. Belief in spiritual-religious treatment	1.729	0.87	3.714	1.147	–1.985	< 0.001***	–1.959
2. Belief in fate/destiny	2.906	1.211	3.705	1.216	–0.799	< 0.001***	–0.658

*p < 0.05, **p < 0.01, ***p < 0.001.

*Phi φ = 0.1 and Cohen’s d between 0 to 0.3 are considered to be small effect.*

*PHQ-15, Patient Health Questionnaire-15; SSD-12, Somatic Symptom Disorder-Criteria B; PANAS PA, Positive Affect; PANAS NA, Negative Affect; STAI Y1, State Anxiety; STAI Y2, Trait Anxiety; SAS, Somatosensory Amplification Scale; SBI, Systems of Belief Inventory.*

In Table 2, Indonesian participants’ responses to the PHQ-15 showed that women more frequently experienced somatic symptoms than men (*p* < 0.01, Cohen’s *d* = −0.304). Additionally, somatosensory amplification in Indonesian women higher than Indonesian men (*p* < 0.01, Cohen’s *d* = −0.343; Table 2). Both differences showed a small effect size. However, there were no significant differences in somatic symptoms and somatosensory amplification between Japanese men and women participants. Mean comparison analysis in Japanese participants found the positive affect in Japanese women higher than Japanese men, but also with small effect size (*p* < 0.01, Cohen’s *d* = −0.279; Table 2).

**TABLE 2:** Comparison between men and women in the Japanese and Indonesian participants.

	Men		Women		*t*-statistics		Cohen’s *d*
			
	Mean	SD	Mean	SD		*P*	
Japan	*n* = 254		*n* = 215				
PHQ > PHQ-15	19.642	5.122	19.116	4.683	1.151	0.250	0.107
SSD > SSD-12	19.984	6.893	20.614	7.164	–0.968	0.333	–0.09
PANAS PA	26.154	8.663	28.479	7.957	–3.006	0.003**	–0.279
PANAS NA	24.677	8.618	25.721	8.067	–1.346	0.179	–0.125
STAI Y1	44.516	10.594	45.093	10.78	–0.583	0.559	–0.054
STAI Y2	49.173	9.679	48.628	9.24	0.621	0.535	0.058
SAS	31.079	6.96	32.284	6.369	1.942	0.053	–0.18
SBI	6.327	6.772	7.619	7.602	–1.946	0.053	–0.18
Indonesia	*n* = 174		*n* = 263				
PHQ	21.144	5.058	22.658	4.941	–3.106	0.002**	–0.304
SSD	27.167	8.915	27.3	9.07	–0.152	0.879	–0.015
PANAS PA	36.477	7.454	35.54	6.292	1.415	0.158	0.138
PANAS NA	37.098	7.07	37.886	6.254	–1.224	0.222	–0.12
STAI Y1	45.787	9.92	46.791	9.288	–1.076	0.283	–0.105
STAI Y2	49.351	8.173	48.631	8.096	0.906	0.365	0.089
SAS	28.483	7.413	31.004	7.292	–3.514	0.001**	–0.343
SBI	31.971	10.014	33.471	8.354	–1.696	0.091	–0.166

*p < 0.05, **p < 0.01, ***p < 0.001.

*PHQ-15, Patient Health Questionnaire-15; SSD-12, Somatic Symptom Disorder-Criteria B; PANAS PA, Positive Affect; PANAS NA, Negative Affect; STAI Y1, State Anxiety; STAI Y2, Trait Anxiety; SAS, Somatosensory Amplification Scale; SBI, Systems of Belief Inventory.*

Furthermore, somatic symptoms for both cultures were positively correlated with somatosensory amplification, health concerns, state anxiety, and trait anxiety (Table 3).

**TABLE 3:** Correlations among related variables for each cultural group.

	1	2	3	4	5	6	7	8
(1). PHQ > PHQ-15	–	0.337**	–0.063	0.010	0.272**	0.331**	0.318**	0.053
(2). SSD > SSD-12	0.391**	–	0.079	0.153**	0.284**	0.342**	0.215**	–0.033
(3). PANAS PA	−0.094*	0.021	–	0.476**	−0.219**	–0.066	0.193**	0.135**
(4). PANAS NA	0.299**	0.237**	0.421**	–	0.027	0.173**	0.295**	0.186**
(5). STAI Y1	0.317**	0.214**	−0.203**	0.430**	–	0.734**	0.009	−0.135**
(6). STAI Y2	0.381**	0.262**	−0.214**	0.466**	0.637**	–	0.208**	−0.142**
(7). SAS	0.188**	0.443**	0.120*	0.246**	0.178**	0.253**	–	0.088
(8). SBI	0.107*	0.089	0.110*	0.079	0.062	0.039	0.001	–

*p < 0.05, **p < 0.01.

*Correlations for Japanese participants are shown below the diagonal (n = 469), and correlations for Indonesian participants (n = 437) are shown above the diagonal. PHQ-15, Patient Health Questionnaire-15; SSD-12, Somatic Symptom Disorder-Criteria B; PANAS PA, Positive Affect; PANAS NA, Negative Affect; STAI Y1, State Anxiety; STAI Y2, Trait Anxiety; SAS, Somatosensory Amplification Scale; SBI, Systems of Belief Inventory.*

### Model of Affective State, Subjective Body Perception, and Spiritual Beliefs With Somatic Symptoms

**FIGURE 1:**
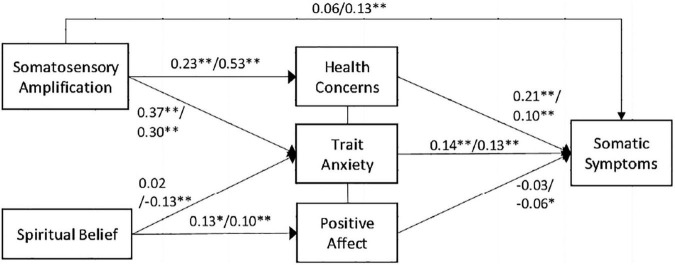
Model of affective state, subjective body perception, and spiritual belief with somatic symptoms. The Japanese participants are mentioned first followed by the Indonesian participants. *p < 0.05, **p < 0.01.

The structural equation modeling (SEM) analysis of the assumed path between somatic symptoms, affective state (i.e., health concerns, trait anxiety, and positive affect), somatosensory amplification, and spirituality exhibited a better fit (Japan: χ^2^ = 25.596, *df* = 3, *p* = 0.000, CFI = 0.910, RMSEA = 0.127; Indonesia: χ^2^ = 12.239, *df* = 3, *p* = 0.007, CFI = 0.967, RMSEA = 0.084) (Figure 1). Moreover, the multigroup model with regression coefficients of two groups was compared. The analysis showed a significant difference, χ^2^_diff_ = 37.836, *df* = 6, *p* = 0.000. Affective states (i.e., health concerns and trait anxiety) were correlated with somatic symptoms (*p* < 0.01). Somatosensory amplification was positively associated with health concerns (*p* < 0.01). Furthermore, trait anxiety was positively related to somatosensory amplification for both cultures (*p* < 0.01).

Meanwhile, in the Indonesian participants, spirituality did not directly link with somatic symptoms. Low levels of spirituality were moderated by high levels of trait anxiety in the association of somatic symptoms. Moreover, high levels of positive affect moderated high levels of spiritual belief to low levels of somatic symptoms only among the Indonesian participants (*p* < 0.05). Among the Japanese participants, no association was observed between spirituality and trait anxiety. Nevertheless, both cultures displayed a positive association between spiritual belief and positive affect (Japan: *p* < 0.05; Indonesia: *p* < 0.01).

## Discussion

This study reports differences in health concerns, positive and negative affect, state anxiety, and spiritual belief between Japanese and Indonesian participants. But, the difference in the somatosensory amplification was inconsiderable. In this study, the differences between men and women in both cultures were also inconsiderable. However, the study presented an association among psychological and spiritual factors in the development of somatic symptoms between the Japanese and Indonesian respondents.

The findings clearly indicated that somatic symptoms emerge from affective state factors. The results were consistent with the relevant works of literature. For example, Maulina (2017) found that trait anxiety (negative emotion level) was consistently higher in individuals with clinically high levels of health anxiety than those without. Moreover, trait anxiety and health anxiety or health concerns strongly influenced somatic symptoms (Pennebaker, 2000; Maulina, 2017). As such, individuals with trait anxiety hold pessimistic views about the world (Pennebaker, 2000). High levels of trait anxiety were linked to high levels of negative affect.

Moreover, Köteles and Simor (2014) found that high levels of health concerns or health anxiety are correlated with high levels of somatosensory amplification. Additionally, high levels of trait anxiety moderate high levels of somatosensory amplification or high levels of bodily symptom sensitivity with somatic symptoms. Thus, individuals with trait anxiety are hypervigilant about their bodies and hold a lower threshold for noticing and reporting subtle physical perceptions (Pennebaker, 2000). They are also likely to worry about the implications of their perceived symptoms.

The bio-psycho-spiritual model reveals the potential pathways between spirituality and pain, because spiritual beliefs may correlate with psychosocial and physiological changes (Wachholtz et al., 2007). This study demonstrated that affective states moderate somatosensory amplification and spiritual belief related to somatic symptoms. The positive cognitive and emotional aspects of spirituality and religion consistently create a buffer against anxiety (Rosmarin and Leidl, 2020). Additionally, among the highest-quality studies that examined the association of spirituality to well-being, happiness, or life, 82% reported positive associations (Koenig, 2018). Spirituality denotes positive emotions, such as love, hope, joy, forgiveness, compassion, trust, gratitude, and awe (Vaillant, 2008, 2013). The effect of positive emotions on the autonomic nervous system has much in common with the relaxation response to meditation (Benson and Stark, 1997). In contrast to the fight-or-flight response induced by negative emotions, which activate the sympathetic nervous system, positive emotion activates the parasympathetic nervous system. Similar to meditation, positive emotions, such as joy, compassion, attachment, trust, and forgiveness, may decrease metabolism, blood pressure, heart rate, respiratory rate, and blood cortisol levels. In the current study, positive emotions decreased somatic symptoms. Spirituality may play an essential part in responding and coping with illness in the clinical setting. A previous study found higher spirituality was significantly associated with lower severity of fatigue and depression in cancer patients (Miller et al., 2022). Meanwhile, in individuals with coronary heart disease (CHD), higher level of spirituality was associated with lower levels of depressive symptoms, less anxiety, and less anger (Ginting et al., 2015). As the need for a new medical model, an integration of biopsychosocial and spiritual suggested by Dyer raised all of the elements. The response variability in spirituality also can be seen personally and are beneficial (Dyer, 2011).

Furthermore, this study found firmer spirituality beliefs among the Indonesian participants than the Japanese participants. In the additional survey, the Indonesians, specifically the university students, believe in fate/destiny and spiritual-religious treatment headed by a religious leader. The beliefs explained that only Indonesia exhibited the role of spirituality in the somatic symptoms model, although the correlation analyses revealed significant positive associations between spirituality and positive affect for both cultures. The beliefs of spirituality in Japan may be unique. The Japanese believe in invisible powers and feel that they can rely on them. Thus, they do not necessarily believe in religion (Nishi, 2009; Nakayama, 2019). In western countries, people tend to become spiritual believers but not religious after denying their faith and taking a stance toward its religious traditions and systems. In Japan, however, spiritual belief is not based on any positive or deliberate rejection of religious establishment or its systems, especially among young people. This group pours into Japanese temples and shrines in search of the so-called *power spots* or *sacred places* to heal or revive visitors, which may influence the human body and mind by producing a form of spiritual energy (Kotera, 2011; Nakayama, 2019).

Japan and Indonesia have an interdependent model of self-construal, which is typical of Asian countries (Singelis, 1994; Park and Kitayama, 2014). This resemblance in culture demonstrates the importance of maintaining harmony in the community. The Japanese culture displays many characteristics, such as prioritizing group harmony over individual opinions, where Japanese people exhibit a strong sense of shame for “losing face.” In addition to maintaining peace, Asians tend to somatize negative experiences more than westerners do (Choi et al., 2016). Specifically, Asians emphasize somatic symptoms instead of emotional states in their communication, which can be understood as their cultural norms for expressing emotions (Kleinman, 1986; Choi et al., 2016). In this study, the Indonesians displayed a higher mean of somatic symptoms and health concerns compared with that of the Japanese. As such, the Japanese are less interdependent than most Asian neighbors, including Indonesians (Country Comparison - Hofstede, 2001; Hofstede Insights, 2021). Indonesia has a lower preference for avoiding uncertainty compared with Japan, which is one of the most uncertainty-avoidant countries. The Japanese have learned to prepare for any uncertain situation and allocate much time and effort into feasibility studies and the examining risk factors. In contrast, Indonesia strongly prefers the separation of the internal self from the external self as practiced in Javanese culture, the largest ethnic group in Indonesia. When a person is upset, Indonesians customarily refrain from expressing negative emotions or anger. Thus, they tend to keep smiling and remain polite regardless of how angry they may be on the inside. Direct communication is frequently perceived as threatening and uncomfortable, which leads them to avoid confrontations. Thus, somatic symptoms tend to emerge due to significant distress or problem in functioning. Spirituality may also facilitate increased tolerance to uncertainty (Rosmarin and Koenig, 2020).

Additionally, as a developing country, community healthcare system in Indonesia is still growing. Alternatively, Japan operates one of the best healthcare systems in the world for various reasons, such as availability, effectiveness, and efficiency. In the past 50 years, Japan has achieved good demographic health at a reasonably low cost (Hamada and Lapalme-Remis, 2008; Hashimoto et al., 2011). This reason may lower the Japanese concerns for health issues.

## Limitations

In this study, spirituality was limited in terms of its associations with other variables, such as social support and the COVID-19 pandemic context. Additionally, this study investigated two cultures only and was limited to the emerging and young adulthood periods. Therefore, further studies should explore the somatic symptoms experienced by other cultures and their association with spirituality beliefs and consider the various developmental stages, e.g., middle adulthood. The participants were recruited from university-based samples of healthy individuals. A clinical setting study with intervention programs may benefit the healthcare system, particularly in patient-doctor interaction. Further cross-cultural research and longitudinal study are required to confirm the current findings and improve their generalizability. Another related issue is the accuracy of self-reported measures; hence, future studies may benefit from incorporating objective physiological method, psychological variables, and spirituality in the experimental setting.

## Conclusion

Comparative studies on understanding somatic symptoms and their relationship with the psycho-spiritual variable in Asian countries are scarce. Despite these limitations, the current study elucidates the association of affective state, subjective body perception, and spirituality with the prevalence of somatic symptoms. Spirituality contributed to the psychological process of somatic symptoms in one culture, wherein positive emotions may moderate spiritual belief to decrease somatic symptoms. In healthy individuals from the two Asian countries, namely, Japan and Indonesia, health concerns and trait anxiety, which were linked to somatosensory amplification, contributed to somatic symptoms.

## Data Availability Statement

The raw data supporting the conclusions of this article will be made available by the authors, without undue reservation.

## Ethics Statement

The studies involving human participants were reviewed and approved by the Ethics Committee of Nagoya University. The patients/participants provided their written informed consent to participate in this study.

## Author Contributions

VM and HO designed the study. VM conducted the research, analyzed the data, and drafted the manuscript. MY collected the data. HO provided the critical feedback. All authors read and approved the final version of the manuscript.

## Conflict of Interest

The authors declare that the research was conducted in the absence of any commercial or financial relationships that could be construed as a potential conflict of interest.

## Publisher’s Note

All claims expressed in this article are solely those of the authors and do not necessarily represent those of their affiliated organizations, or those of the publisher, the editors and the reviewers. Any product that may be evaluated in this article, or claim that may be made by its manufacturer, is not guaranteed or endorsed by the publisher.
