# Corrigendum: Somatic symptoms: association among affective state, subjective body perception, and spiritual belief in Japan and Indonesia

**DOI:** 10.3389/fpsyg.2024.1516816

**Published:** 2024-11-19

**Authors:** Venie Viktoria Rondang Maulina, Masao Yogo, Hideki Ohira

**Affiliations:** ^1^Department of Cognitive and Psychological Sciences, Nagoya University, Nagoya, Japan; ^2^Department of Psychology, Atma Jaya Catholic University of Indonesia, Jakarta, Indonesia; ^3^Department of Psychology, Doshisha University, Kyoto, Japan

**Keywords:** somatic symptoms, health concerns, trait anxiety, positive affect, negative affect, somatosensory amplification, spirituality

In the published article, there was an error in the **Ethics Statement**. The statement previously stated, “The studies involving human participants were reviewed and approved by the Ethics Committee of Nagoya University. The patients/participants provided their written informed consent to participate in this study”. The correct Ethics Statement appears below.

